# Oral Administration of Alpha Linoleic Acid Rescues Aβ-Induced Glia-Mediated Neuroinflammation and Cognitive Dysfunction in C57BL/6N Mice

**DOI:** 10.3390/cells9030667

**Published:** 2020-03-09

**Authors:** Waqar Ali, Muhammad Ikram, Hyun Young Park, Min Gi Jo, Rahat Ullah, Sareer Ahmad, Noman Bin Abid, Myeong Ok Kim

**Affiliations:** 1 Division of Applied Life Science (BK 21), College of Natural Sciences, Gyeongsang National University, Jinju 52828, Korea; waqarali_93@gnu.ac.kr (W.A.); qazafi417@gnu.ac.kr (M.I.); mingi.cho@gnu.ac.kr (M.G.J.); rahatullah1414@gnu.ac.kr (R.U.); sareer_50@gnu.ac.kr (S.A.); noman_abid@gnu.ac.kr (N.B.A.); 2 Maastricht University Medical Center (MUMC+), School for Mental Health and Neuroscience|Alzheimer Center Limburg, 6229ER Maastricht, The Netherlands; hailey.park@maastrichtuniversity.nl

**Keywords:** Alzheimer’s disease, neuroinflammation, neurodegeneration, omega-3 fatty acids

## Abstract

In this work, we evaluated the effects of alpha linoleic acid (ALA), an omega-3 polyunsaturated fatty acid, on amyloid-beta-induced glial-cell-mediated neuroinflammation, amyloidogenesis, and cognitive dysfunction in mice. After an infusion of Aβ_1–42_ (Aβ_1–42_, 5 μL/5 min/mouse, intracerebroventricular injection (i.c.v), and respective treatments of ALA (60 mg/kg per oral for six weeks), neuroinflammation, apoptotic markers, and synaptic markers were evaluated by Western blot and immunofluorescence analyses. According to our findings, the infusion of Aβ_1–42_ activated Toll-like receptor 4 (TLR4), glial fibrillary acidic protein (GFAP), and ionized calcium adaptor molecule 1 (Iba-1) in the frontal cortices and hippocampi of the Aβ_1–42_-injected mice to a greater extent than the Aβ_1–42_ + ALA-cotreated mice. Similarly, there was an elevated expression of phospho-c-Jun-N-terminal kinase (p-JNK), phospho-nuclear factor-kB p65 (p-NF-kB p65 (Ser536)), and tissue necrosis factor (TNF) in the Aβ_1–42_ infused mouse brains; interestingly, these markers were significantly reduced in the Aβ + ALA-cotreated group. The elevated expression of pro-apoptotic markers was observed during apoptotic cell death in the Aβ_1–42_-treated mouse brains, whereas these markers were markedly reduced in the Aβ + ALA-cotreated group. Moreover, Aβ_1–42_ infusion significantly increased amyloidogenesis, as assessed by the enhanced expression of the amyloid precursor proteins (APP) beta-amyloid cleaving enzyme-1 (BACE-1) and amyloid-beta (Aβ_1–42_) in the mouse brains, whereas these proteins were markedly reduced in the Aβ + ALA-cotreated group. We also checked the effects of ALA against Aβ-triggered synaptic dysfunction and memory dysfunction, showing that ALA significantly improved memory and synaptic functions in Aβ-treated mouse brains. These results indicated that ALA could be an applicable intervention in neuroinflammation, apoptotic cell loss, amyloidogenesis, and memory dysfunction via the inhibition of TLR4 and its downstream targets in Aβ + ALA-cotreated mouse brains.

## 1. Introduction

Alzheimer’s disease (AD) is a slowly progressive, chronic neurological disease characterized by the loss of memory and cognition [1,2]. The exact mechanism of AD is unknown, but genetic [3] and environmental factors, such as aging, family history, comorbidities such as diabetes and cardiovascular disease [4,5], a substandard lifestyle, and nutrition are thought to play roles in its spread. Besides the accumulation of amyloid-beta (Aβ_1–42_) plaques and hyperphosphorylation of neurofibrillary tangles (NFTs), which are considered to be the two main hallmarks of AD, other contributors to disease onset include oxidative stress [6], neuroinflammation, and apoptotic cell death [7].

Studies have suggested that Alzheimer’s disease pathogenesis is not confined only to neuronal cells but is also strongly linked to the brain’s immune system. Aggregated and misfolded proteins bind to pattern recognition receptors on astromicroglia and elicit an innate immune reaction categorized by a release of neuroinflammatory inducers, which are associated with neurodegenerative diseases’ progression [8]. Microglial cells are the resident phagocytic cells of the central nervous system (CNS), and their phagocytic capacity can be modulated to improve the clearance of Aβ deposition. This indicates that inhibiting the microglial phenotype, thereby switching it from the pathogenic state to the normal cognitive phenotype, or specifically activating microglia could comprise a promising and effective therapeutic approach for AD-like neurodegenerative conditions [9]. Neuroinflammation following the activation of the immune system leads to neurodegeneration in specific regions of the central nervous system (CNS). Therfore, adequate regulation of the immune response within the CNS is critical, because most of the brain diseases are related to chronic inflammatory conditions and accompanied by microglia activation that tracks inappropriate T cell instigation and polarization. Brain immune cells and microglia are sensitive to intrinsic and extrinsic provocations that control neuroinflammation, thereby helping to combat pathogenic/injurious stimulation and start the healing processes observed in neurological conditions [10,11]. During acute brain injury, chronic neuroinflammation accelerates neurodegeneration and contributes to the pathogenesis of Parkinson’s, Alzheimer’s disease, Huntington’s disease, multiple sclerosis, and other cognitive and memory dysfunctions [12,13]. The release of brain inflammatory cytokines may be facilitated by many factors, including pattern recognition receptors (PRRs), and more specifically Toll-like receptor-4 (TLR4).

Microglia that express TLR4 mediate neuronal cell loss [14]. Microglia activation has been reported in various neurodegenerative conditions, while its inhibition/suppression was found to protect against neurodegeneration [15]. Similarly, alterations in TLR4 receptors suppressed microglial instigation and preserved cognitive function in a mouse model of Alzheimer’s disease. TLR4 is a pattern recognition receptor exhibited by different cell types, including macrophages, microglial cells, astrocytes, and neuronal cells [13], and it was shown to have modulatory effects in the adrenal response to inflammation and stress [14,15]. Provocation of the TLR4 complex may trigger the pathophysiology of various neurological disorders, including Alzheimer’s and Parkinson’s, and inhibition of the TLR4 receptor may alleviate NF-κB (nuclear factor-kB p65) activation and the suppression of pro-inflammatory cytokines both in the prefrontal cortices and hippocampi of experimental mice [16]. Apoptotic cell death is another factor that plays a role in Aβ_1–42_-induced neurotoxicity, which is also promoted by various mediators, such as Jun-N-terminal kinase (JNK) [17], and activation of the innate immune system, which is responsible for neurodegeneration and memory loss [18]. The main markers, including caspases, BCL2-associated X protein (Bax), and poly-ADP-ribosyltransferase (PARP-1), are implicated in synaptic dysfunction, memory impairment, and neurodegeneration [19,20].

Alpha linoleic acid is an omega-3 polyunsaturated fatty acid obtained from various plant sources, such as walnuts, flaxseed oil, soybean oil, and many more [21]. Alpha linoleic acid (ALA) has roles in brain development and function, inflammatory responses, antioxidative activities [22]. Moreover, studies suggested that ALA improves the cognitive dysfunction caused by Aβ_1–42_ in a mouse model of AD [23] and inhibits the proliferation of inflammatory cytokines, inducible nitric oxide synthase (iNOS) [24], and cyclooxygenase-2 (COX-2) in Aβ_1–42_-induced AD models [25]. ALA upregulates Aβ_1–42_-degrading enzymes (IDE), thereby preventing neuronal necrosis and apoptosis [26]. ALA has strong free radical scavenging and antioxidative propeties to attenuate inflammatory responses and prevent cellular damage and apoptosis [27]. Based on the potential neuroprotective effects of ALA against different neurodegenerative conditions as described previously, we hypothesized that ALA abrogates Aβ_1–42_-induced neuroinflammation via the inhibition of TLR4, glial fibrillary acidic protein (GFAP), and ionized calcium adaptor molecule 1 (Iba-1) expression. For this, we developed an animal model of Alzheimer’s disease via intracerebroventricular (i.c.v) injection of amyloid-beta peptides. The amyloid fibrils associated with AD were formed from the Aβ peptide, which occurs in different isoforms of varrying lengths. The 40-residue peptide Aβ (1–40) represents the highly expressed Aβ isoform in the brain, while the 42-residue Aβ (1–42) represents the most expressed isoform in certain forms of AD cases [28]. The Aβ42 isoform aggregates to form small dimers, trimers, oligomers, protofibrils, and large insoluble fibrils [29]. To demonstrate almost all amyloid beta forms, we injected the Aβ_1–42_ peptides into the mouse brains, as this method produces the most accepted and well-established model. For further analysis, we conducted Western blot and immunofluorescence studies for glial cell-mediated neuroinflammation, apoptotic cell death, and memory-related markers in brain samples of the experimental groups.

## 2. Materials and Methods

### 2.1. Experimental Animals

Male C57BL/6N mice (8 weeks old, *n* = 64, 16 mice per group) weighing between 24 and 30 g were purchased from Samtako Bio, Osan, South Korea. The animals were handled in accordance with the procedures of the Institutional Animal Care and Use Committee (IACUC) of the Division of Applied Life Science, Gyeongsang National University, South Korea (Approval ID: 125). The mice were bred for 7 days in an animal care house (4–5 per cage) under standard environmental conditions (temperature, 20 ± 2 °C; humidity 40% ± 10%; 12 h light/dark cycle), and were provided with normal pellet food and water ad labium.

### 2.2. Mice Grouping and Treatment

The mice were separated into four different groups (*n* = 16 per group): the control group (i.c.v 0.9% NaCl), the injected + oral water administered Aβ-infused mice group (i.c.v. injected Aβ_1–42_), the Aβ_1–42_ + alpha linoleic acid-treated group (Aβ_1–42_ + ALA, 60 mg/kg/day), and the alpha linoleic acid alone group (ALA, 60 mg/kg/day/per oral (p.o.). ALA was given orally (P.O.) for 6 weeks (one week post-Aβ_1–42_ injection and for 5 weeks after Aβ_1–42_) using a curved oral gavage. The alone group was included to show any unwanted effects associated with the dissolution of ALA in ethanol.

### 2.3. Intracerebroventricular (ICV) Injection of Amyloid-β Peptides

For the injection of the Aβ_1–42_ peptide, previously published protocols were followed, with minor changes whenever necessary [30]. The human Aβ_1–42_ peptide was made up as a stock solution (1 mg/mL in sterile saline solution), followed by incubation at 37 °C for four days. The aggregated form of the Aβ_1–42_ peptides (5 μL/mouse) or the respective vehicles (0.9% NaCl, 5 μL/mouse) were stereotaxically injected into the ventricles using a Hamilton microsyringe (−0.2 mm anteroposterior (AP), 1 mm mediolateral (ML), and −2.4 mm dorsoventral (DV) into the bregma) under anesthesia in combination with 0.05 mL/100 g bodyweight of Rompun (xylazine) and 0.1 mL/100 g bodyweight of Zolitil (ketamine) at a rate of 1 μL/5 min. The injector was left in the injection site for 5 min, as suggested previously [31].

### 2.4. Preparation of Alpha Linoleic Acid for Oral Administration

The ALA was dissolved in a small amount of ethanol, and the final volume was adjusted with normal saline. To prevent oxidation, the solution was stored in a light-proof amber glass bottle and a small amount of 1,4-dithiothreitol (DTT) was added.

### 2.5. Behavioral Studies

After the completion of the respective treatments, behavioral tests were conducted, including the Morris Water Maze (MWM) and the Y-Maze tests [32]. The apparatus used for the MWM test was used to evaluate the memory and learning performance of mice; it was made of a circular water tank with a diameter of 100 cm and a height of 35 cm. It was filled with white-colored water and maintained at 23 °C with a depth of 15.5 cm [33]. A hidden platform (4.5 cm in diameter and 14.5 cm in height) was placed in one quadrant of the apparatus, 1 cm below the water surface. A mouse was placed in one quadrant and allowed to find the hidden platform [34]. All of the mice individually received four training trials for five consecutive days. The time spent by each mouse to find the submerged platform for each trial was recorded. On day 6, a probe test was conducted by removing the hidden platform and allowing the mice to freely swim for 1 min. The time spent by the mice in the target quadrant (where the hidden platform was located during training) and the number of crossings of the mice over the location of the hidden platform were calculated [35]. The behavioral data were recorded using a video camera.

For the evaluation of spatial working memory, the Y-Maze assembly was used, which was made of three arms and arranged in a Y-shaped manner with dimensions of 30 × 15 × 15 cm, with an indicator at the top [36]. All of the mice were placed in the middle of the maze individually and allowed to move freely for different intervals of time (3–8 min). Glowing indicators confirmed when each mouse entered one of the arms. The entry of mice into three different arms in overlapping triplets was considered to be spontaneous alternation [32]. The percentage (%) of alternation behavior was calculated as the number of successive entries into three different arms consecutively/total number of arm entries 2 × 100. A greater percentage of spontaneous alternation behavior reflected improved cognitive function [37].

### 2.6. Immunobloting

After behavioral analyses, the mice were anesthetized via a combination of ketamine and xylazine and euthanized, as previously described [6,38]. The brain parts were separated and homogenized in PRO-PREP^TM^ extraction solution (iNtRON Biotechnology, Inc., Sungnam, South Korea) and centrifuged (13,000 RPM for 25 min at 4 °C). The supernatant was collected and stored at −70 °C for further studies. The concentrations of the protein samples were calculated using a Bradford assay (Bio-Rad Protein Assay kit, Bio-Rad Laboratories, Pleasanton, CA, USA). The samples were separated on 12.5% sodium dodecyl sulfate (SDS-PAGE GEL) (Merck KGaA, Darmstadt, Germany) and transferred to a polyvinylidene fluoride (PVDF) membrane (Immobilon-PSQ, transfer membrane, Merck Millipore, Burlington, MA, USA). The membranes were blocked with 5% skim milk for 1 h at room temperature (25 °C). The membranes were incubated with the required primary antibodies (diluted in 1X Tris-Buffered Saline, 0.1% Tween® 20 Detergent (1×TBST) at 4 °C for 24 h and washed with 1×TBST (3 times for 10 min), then reacted with appropriate secondary antibodies (anti-rabbit/anti-mouse, diluted in TBST) for 2 h at room temperature and washed with TBST. The expressions of the bands were captured on X-ray films using enhanced chemiluminescence (ECL) reagent (ATTO Corporation, Tokyo, Japan). The Western blot results (minimum of three results for each marker) were quantified using Image J (v. 1.50, NIH, Bethesda, MD, USA), and the expressions of the bands were normalized to the respective control groups. β-actin was used as a loading control. The data were presented in terms of fold change, and the graphs were generated using GraphPad Prism v6 software (GraphPad Software, San Diego, CA, USA).

### 2.7. Chemicals and Antibodies

Alpha-linoleic acid (cis-9,cis-12-octadecadienoic acid, Lot#30H8479) and Aβ_1–42_ peptides were purchased from Sigma-Aldrich Chemical Co., St. Louis, MO, USA. The antibodies used in the study included anti-Aβ (sc-28365), anti-p-Tau (sc-390476), anti-BACE-1 (sc-33711), p-Tau (sc-)anti-PSD-95 (sc-71933), anti-synaptosomal-associated protein 23 (SNAP-23) (sc-374215), anti-p-JNK (sc-6254), anti-Caspase 3 (sc-7272), anti-Bax (sc-7480), anti-Bcl-2 (sc-7382), anti-PARP-1 (sc-7008), anti-TNF-α (sc-52746), anti-p-NF-κB p65 (Ser536) (sc-136548), anti-TLR4 (sc-293072) anti-Iba-1 (sc-32725), anti-GFAP (sc-33673), and anti-β-actin (sc-47778) from Santa Cruz Biotechnology (Dallas, TX, USA). The primary antibodies were diluted in TBST (1:1000) (Santa Cruz Biotechnology) and the secondary anti-mouse HRP conjugated (Promega Ref# W402) and antirabbit HRP conjugated (Promega Ref# W401) antibodies that were diluted to 1:10,000 in 1 × TBST were obtained from Promega, USA. For confocal microscopic studies, the secondary fluorescent antibodies used were goat anti-mouse (Ref# A11029) and goat anti-rabbit (Ref# 2732), which were diluted in 1× phosphate-buffered saline (PBS).

### 2.8. Tissue Preparation for Immunofluorescence Analyses

For immunofluorescence analyses, the mice were euthanized (with ketamine and xylazine) and perfused transcardially with 0.1 M phosphate-buffered saline (PBS) and 4% neutral buffered formalin and frozen in (Optimal Cutting Temperature) OCT compound; then, they were cut into uniform sections (14 μm) using a microtome (Leica CM 3050C, Burladingen, Germany), as performed previously. The sections were thaw-mounted on Probe-On Plus charged slides (Fisher, Rockford, IL, USA) and stored at −70 °C.

### 2.9. Immunofluorescence Staining

Immunofluorescence staining was carried out according to standard protocols [39,40]. The slides were dried at room temperature overnight and washed with 0.1 M PBS two times for 10 min. Then, the slides were incubated with proteinase K for 5 min and washed two times for 5 min. The slides were blocked with 2% normal goat serum (rabbit/mouse) and 0.1% Triton X-100 in 0.1 M PBS for 1 h. Then, the slides were incubated with primary antibodies overnight at 4 °C, washed with 0.1 M PBS, and treated with fluorescein isothiocyanate (FITC)-labeled (green) or tetramethylrhodamine (TRITC)-labeled (red) secondary antibodies (anti-mouse and anti-rabbit) at RT for 90 min. Finally, the slides were counterstained with 4′,6-diamidino-2-phenylindole dihydrochloride (DAPI) for 8 min and covered with fluorescent mounting medium using glass coverslips. The images were captured using a confocal laser-scanning microscope (FluoView FV 1000 MPE). A minimum of six pictures was taken for each marker and analyzed using Image J software (v. 1.50, NIH), and the intensities were compared with the other groups. DAPI was used to visualize the nuclear DNA in the fixed neuronal cells. The pictures were taken with the same exposure time and brightness/contrast parameters and normalized to the number of DAPI-stained cells.

### 2.10. Statistical Analyses

ImageJ software was used to measure the relative values to arbitrary units (AU) and integrated density to AU, respectively; the data are presented as the mean ± SEM (three independent experiments for 16 mice per group: 8 for immunofluorescence and 8 for Western blot) and the graphs were generated by using GraphPad Prism v6 software (GraphPad Software) and one-way ANOVA followed by Bonferroni’s multiple comparisons test, which was used for the statistical analysis between the different groups, where *p* < 0.05 was considered significant. * Significantly different from the vehicle-treated group; # significantly different from the Aβ-injected group. Significance = * p < 0.05; ** p < 0.01; # *p* < 0.05; and ## *p* < 0.01.

## 3. Results

### 3.1. Alpha-Linoleic Acid May Reduce TLR4, Activated Astrocyte, and Microglial Marker Expressions in Aβ-Injected Mouse Brains

**Figure 1:**
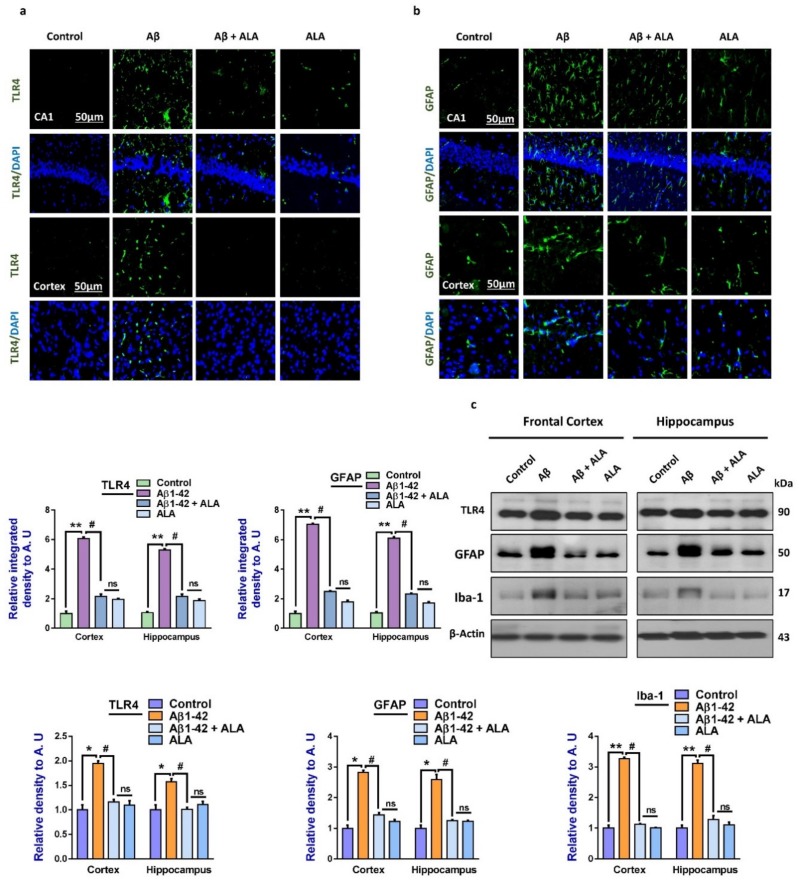
Effects of alpha linoleic acid against amyloid-beta (Aβ)-induced activated Toll-like receptor 4 (TLR4), glial fibrillary acidic protein (GFAP), and ionized calcium adaptor molecule 1 (Iba-1) in mouse brains. (**a**) and (**b**): Immunofluorescence results of TLR4 and GFAP in the frontal cortices and hippocampi (CA1) of the treated mice groups, with respective bar graphs. Magnification: 30×; scale bar: 30 and 50 µm. (**c**) Western blot results of Toll-like receptor 4 (TLR4), glial fibrillary acidic proteins (GFAP), and ionized calcium-binding adaptor molecule 1 (Iba-1), with respective bar graphs. * Significantly different from the Aβ-treated group; # significantly different from the Aβ + ALA-cotreated group. Significance = * p < 0.05; ** p < 0.01; # *p* < 0.05; and ## *p* < 0.01. Aβ: amyloid beta; ALA: alpha linoleic acid; DAPI: 4′,6-diamidino-2-phenylindole; ns = non significant.

We evaluated TLR4 and glial fibrillary acid protein (GFAP) expression levels in the brains of the treated groups through immunofluorescence analysis, as these are considered to be the crucial mediators of neurodegenerative conditions [17]. Enhanced expression levels of TLR4 and GFAP in the Aβ-injected mouse brains (frontal cortices and hippocampi) were observed compared to the saline-injected mouse brains. The elevated expressions of these markers were significantly reduced in the Aβ + ALA-cotreated mouse brains. The Western blot analysis confirmed that that there were enhanced expression levels of TLR4, GFAP, and Iba-1in the Aβ-treated mouse brains, which were significantly lower in the Aβ + ALA-cotreated group, as shown in Figure 1.

### 3.2. Alpha Linoleic Acid Regulates the Expression of p-JNK and Its Downstream Targets in Aβ_1–42_-Treated Mouse Frontal Cortices and Hippocampi

**Figure 2:**
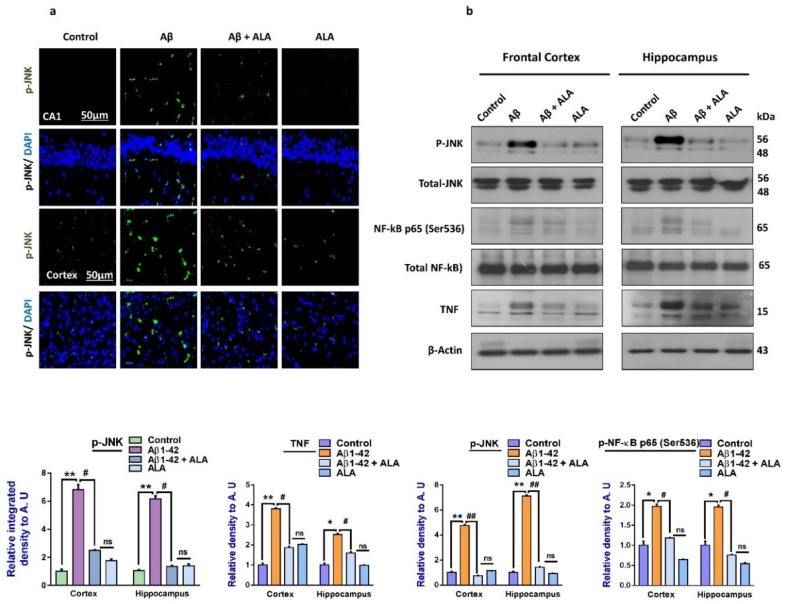
Effects of alpha linoleic acid against Aβ-induced activated p-JNK, p-NF-kB, and TNF-α in mouse brains. (**a**) Immunofluorescence results of p-JNK in the frontal cortices and hippocampi (CA1) of the treated mice groups (*n* = 10 mice per group) with respective bar graphs. Magnification: 30×; scale bar 50 µm. (**b**) Western blot results of p-JNK, p-NF-kB, and TNF-α in the experimental groups with respective bar graphs. * Significantly different from the Aβ-treated group; # significantly different from the Aβ + ALA-cotreated group. Significance = * p < 0.05; ** p < 0.01; # *p* < 0.05; and ## *p* < 0.01. Aβ: amyloid beta; ALA: alpha linoleic acid; DAPI: 4′,6-diamidino-2-phenylindole; ns = non significant.

To evaluate the expression of p-JNK and its downstream targets in the Aβ_1–42_-treated mouse brains, we performed Western blotting for phospho-c-Jun-N-terminal kinase (p-JNK), p-NF-κB, and tissue necrosis factor (TNF)-α in the brains (frontal cortices and hippocampi) of the experimental groups. Our results showed significantly increased expression levels of various inflammatory biomarkers (i.e., p-JNK, p-NF-κB, and TNF-α) in the Aβ_1–42_-treated group compared to the saline-injected control group. Notably, these markers were significantly reduced in the Aβ + ALA-cotreated group. These effects were further confirmed by the immunofluorescence results, which showed an enhanced expression of p-JNK in the frontal cortices and CA1 region of the hippocampi compared to the control group; interestingly, these markers were significantly reduced in the Aβ + ALA-cotreated group, as shown in Figure 2.

### 3.3. Alpha Linoleic Acid May Rescue Mouse Brains Against Aβ_1–42_-Induced Apoptotic Cell Death and Neurodegeneration

**Figure 3:**
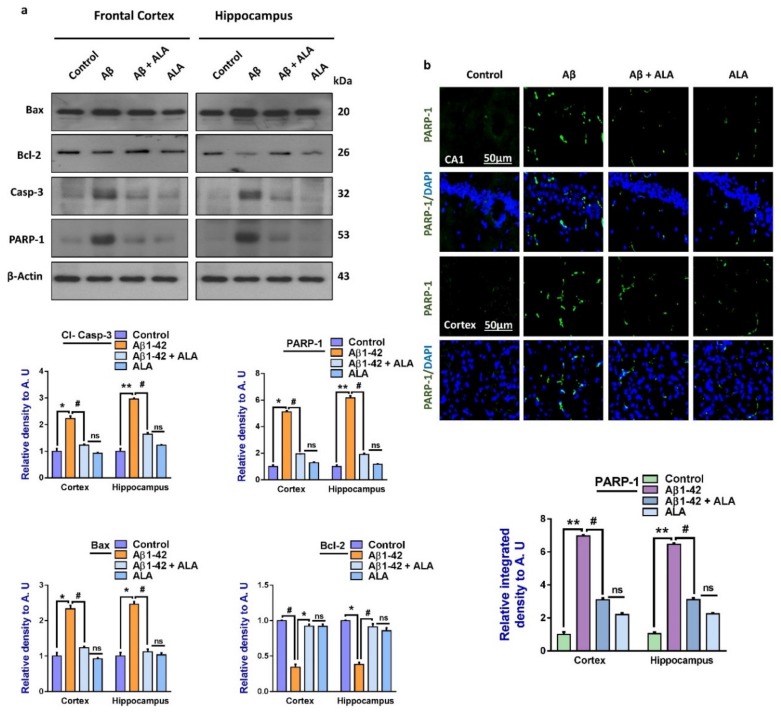
Effects of alpha linoleic acid against Aβ-induced apoptotic cell death in mouse brains. (**a**) Immunoblot results of BCL2-associated X protein (Bax), Bcl-2, caspase-3, and poly-ADP-ribosyltransferase (PARP-1) in the frontal cortices and hippocampi (CA1) of the treated mice groups (*n* = 16 mice per group, 8 for immunofluorescence and 8 for Western blot) with respective bar graphs. (**b**) Immunofluorescence results of PARP-1 in the frontal cortices and hippocampi of the experimental groups (*n* = 10 mice per group) with respective bar graphs. Magnification: 30×; scale bar: 50 µm. * Significantly different from the Aβ-treated group; # significantly different from the Aβ + ALA-cotreated group. Significance = * p < 0.05; ** p < 0.01; # *p* < 0.05; and ## *p* < 0.01. Aβ: amyloid beta; ALA: alpha linoleic acid; DAPI: 4′,6-diamidino-2-phenylindol; ns = non significant.

Another main hallmark of Aβ-induced neurodegeneration is apoptotic cell death [41,42]. To show that overall apoptotic cell death was markedly reduced with ALA administration, we elevated the expression of the pro-apoptotic markers Bax, caspase-3, and PARP-1, and the anti-apoptotic marker Bcl-2, in the experimental groups [30]. The immunofluorescence results were in accordance with the Western blot results, which showed elevated levels of PARP-1 in the Aβ-treated mice brains compared to the saline-injected control mice. Interestingly, the expression of PARP-1 was markedly reduced in the Aβ_1–42_ + ALA-treated mouse brains. The overall findings supported the notion that the TLR4 receptor was counteracted by using ALA, which rescued the mouse brains against Aβ-induced apoptotic cell death and neurodegeneration (Figure 3).

### 3.4. Alpha Linoleic Acid may Rescue Alzheimer’s Disease-Like Pathological Changes in Mouse Brains

**Figure 4:**
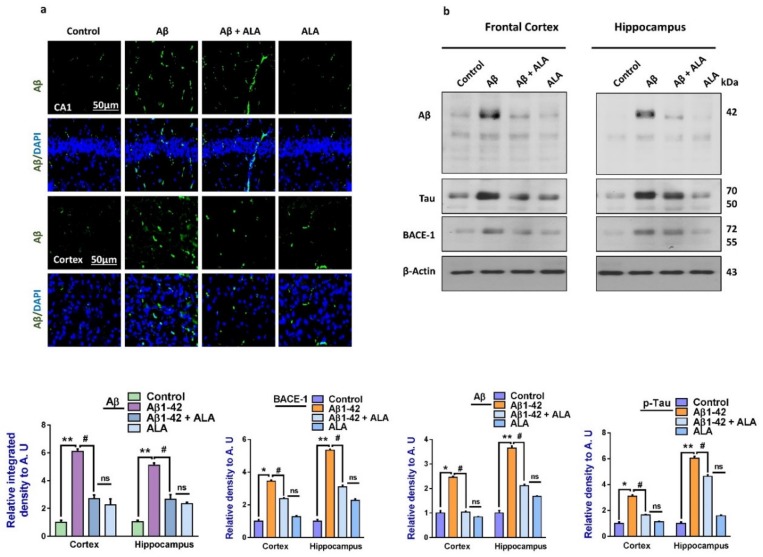
Effects of alpha linoleic acid against Aβ-induced AD-like changes in the mouse brains. (**a**) Immunofluorescence results of Aβ in the frontal cortex and hippocampus of the experimental groups (*n* = 16 mice per group, 8 for IF and 8 for WB), with their respective bar graphs, magnification 30×, scale bar 50 µm. (**b**) Immunoblots results of Aβ, BACE-1, and p-Tau in the frontal cortex and hippocampus (CA1) of the treated mice groups (*n* = 10 mice per group), with respective bar graphs. * Significantly different from the Aβ-treated group; # significantly different from the Aβ + ALA-cotreated group. Significance = * p < 0.05; ** p < 0.01; # *p* < 0.05; and ## *p* < 0.01. Aβ: amyloid beta; ALA: alpha linoleic acid; DAPI: 4′,6-diamidino-2-phenylindole; ns = non significant.

To analyze the effects of ALA on Aβ-induced amyloidogenesis, we analyzed the expression of APP, BACE-1 Aβ, and p-Tau (a marker of Tau hyperphosphorylation) in the frontal cortex and hippocampus of the treated mice groups. According to our findings, there was an enhanced expression of amyloid precursor protein (APP), beta-amyloid cleaving enzyme-1 (BACE-1), Aβ, and p-Tau in the Aβ-injected mouse brains, compared to the saline-treated control group. Interestingly, these markers were significantly reduced in the Aβ + ALA-cotreated group. Furthermore, these results were supported by immunofluorescence analyses, which showed increased immunoreactivity of Aβ in the Aβ-injected mouse brain sections, compared to the saline-treated control groups. Interestingly, the expression of Aβ was significantly reduced in the Aβ + ALA-treated mouse frontal cortex and hippocampus (CA1 region) as shown in Figure 4.

### 3.5. Alpha Linoleic Acid May Regulate Synaptic and Cognitive Functions in Aβ_1–42_-Treated Mouse Brains

**Figure 5:**
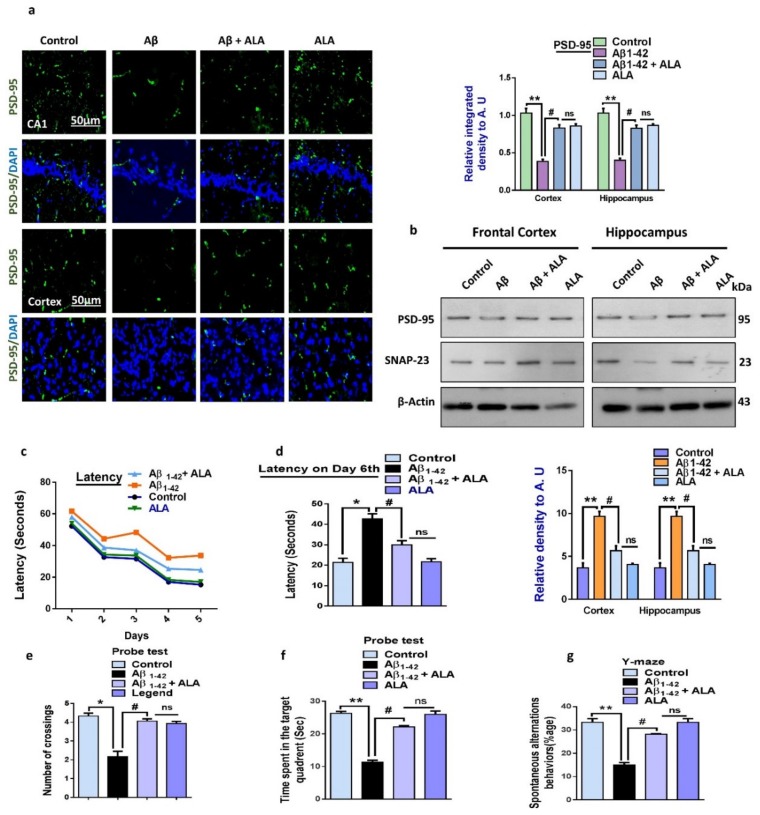
Effects of alpha linoleic acid against Aβ-induced synaptic and memory dysfunctions in mice. (**a**) Immunofluorescence results of PSD-95 in the frontal cortices and hippocampi of the experimental groups (*n* = 10 mice per group) with respective bar graphs. Magnification: 30×; scale bar: 50 µm. (**b**) Immunoblot results of PSD-95 and SNAP-23 in the frontal cortices and hippocampi (CA1) of the treated mice groups (*n* = 16 mice per group, 8 for IF and 8 for WB) with respective bar graphs. (**c**) Bar graph showing the mean time taken to escape (sec) during the training days. (**d**) Final escape time during the probe test. (**e**) Number of platform crossings. (**f**) Time spent in the target quadrant. (**g**) Spontaneous alternation behavior. * Significantly different from the Aβ-treated group; # significantly different from the Aβ + ALA-cotreated group. Significance = * p < 0.05; ** p < 0.01; # *p* < 0.05; and ## *p* < 0.01. Aβ: amyloid beta; ALA: alpha linoleic acid; DAPI: 4′,6-diamidino-2-phenylindole; ns = non significant.

To analyze the effects of ALA in the Aβ_1–42_-treated mouse groups, we assessed various synaptic protein biomarkers, such as SNAP-23, PSD-95, etc. The Western blot results showed a reduced expression of SNAP-23 and PSD-95 in the Aβ_1–42_-treated group compared to the saline-injected control group. Notably, the expression of synaptic markers was markedly upregulated in the Aβ_1–42_ + ALA-cotreated group, indicating that the synaptotoxicity effects of Aβ_1–42_ were reversed. In addition to the Western blot results, according to the immunofluorescence findings, PSD-95 expression was reduced in Aβ_1–42_-treated mouse brains compared to the saline-injected control group. Notably, the immunofluorescence of PSD-95 was markedly elevated in the Aβ_1–42_ + ALA-cotreated group. Moreover, according to the MWM test results, the Aβ_1–42_-treated mice took significantly longer to reach the escape platform compared to the control group, with the Aβ_1–42_ + ALA-cotreated group taking the longest time. Similarly, the Aβ_1–42_-treated mouse group spent less time in the targeted quadrants and crossed the platform less compared to the saline-injected control group. Interestingly, the time spent in the target quadrant and the number of platform crossings were significantly greater in the Aβ_1–42_ + ALA-cotreated group, showing improved memory and cognition. Y-Maze tests were performed to assess spatial working memory using spontaneous alternation behaviors, showing that the Aβ_1–42_-treated mice had a lower percentage of alternation compared to the control group. Notably, the percentage of alternation behavior significantly increased in the Aβ_1–42_ + ALA-cotreated mice, showing that the ALA improved short-term memory dysfunction in the Aβ-injected AD mouse model (Figure 5).

## 4. Discussion

The main findings of the current study were that the long-term oral administration of ALA may relieve Aβ-induced glial cell-mediated neuroinflammation, apoptotic cell death, and synaptic dysfunction in a mouse model of Alzheimer’s disease (AD) by regulating TLR4, p-JNK, p-NF-kB, and p65 (Ser536) expression alongside the release of other inflammatory cytokines.

Previous research studies extensively highlighted inflammation as a crucial player in cardiovascular disease [43] and neurodegenerative conditions [6,44], specifically AD [17] and PD [45]. Here, we found that the chronic oral administration of ALA may inhibit Aβ-induced neuroinflammation by regulating the levels of activated TLR4 and its downstream targets, such as GFAP and Iba-1. Moreover, ALA has strong regulating effects against p-JNK, p-NF-kB p65 (Ser536), and TNF release. Innate immune system activation was followed by the upregulation of TLR4, astrocytes, and microglia in Aβ-injected mouse models and in vitro cells in accordance with previously published reports [17]. Other research studies suggested that TLR4 is involved in many inflammatory diseases such as influenza, cancer, diabetes, and neurodegenerative diseases, thereby suggesting that its regulation (antagonism/inhibition) would have an impressive clinical impact. TLR4 recognizes endogenous molecules such as β-amyloid and α-synuclein, thereby instigating adaptive and innate immune function [38,46].

The TLR system identifies various pathogen-derived and tissue damage-associated ligands. TLR signaling was shown to add to the progression of age-related neurodegenerative conditions, including Alzheimer’s disease [17]. Moreover, TLR4 antagonism supresses the LPS-triggered release of TNF and interleukin-1 beta (IL-β), highlighting the critical role of TLR4 inhibition in inflammatory conditions associated with TLR4 activation [47]; some TLR4 antagonists are currently being tested in clinical trials [48]. Our previous studies also suggested that TLR4 inhibition in Aβ-treated mice (using a plant-derived flavonoid) protects mouse brains against neuroinflammation [17]. Similarly, other studies suggested that the inhibition of TLR4 is protective in LPS-injected mice and BV-2 microglial cells [49]. Here, we found a marked decrease in the levels of TLR4, GFAP, and Iba-1 in the Aβ_1–42_ + ALA-cotreated group. The inhibition of the innate immune response and suppression of TLR4 by ALA administration was in accordance with the previously acknowleged effects of omega-3 polyunsaturated fatty acids [50].

Another main component of neuroinflammation is the phosporylation of JNK, which occurs in many neurodegenerative conditions including Aβ-induced neurodegeneration; the inhibition of phosporylation JNK offers a potential neuroprotective role in traumatic brain injuries and AD models. JNK also plays a critical role in linking all three of the main pathological hallmarks of AD, including the formation of amyloid plaques and neurofibrillary tangles and atrophy of the brain. The phosphorylation of JNK leads to the activation of transcription-related factors that regulate apoptosis, neuronal loss [51], and ultimately cell death in multiple neurodegenerative diseases [52]. Furthermore, JNK phosporylation may elicite the amyloid β (Aβ) peptides [53], and it was also suggested to control the activation of amyloid precursor protein (APP) and ultimately the accumulation of Aβ in the brains [54]. Moreover, strong evidence suggested that the activation of JNK may induce the activation of tau proteins in vitro [55]. In our results, the phosporylation of JNK, NF-kB p65 (Ser536), and TNF-α was increased in the mouse brains; the expression levels of these proteins were markedly reduced with the administration of ALA. The inhibitory effects of ALA against activated JNK, p-NF-kB p65 (Ser536), and TNF were in accordance with previous reports [56].

We also checked the anti-apoptotic effects of ALA. As expected, apoptotic markers were significantly elevated and anti-apoptotic marker expression was significantly reduced, which may be partly due to the upregulation of p-JNK, as mentioned previously [57]. Similarly, the upregulation of TLR4 and subsequent activation of astromicroglial cells may also contribute to the induction of apoptotic cell death and neurodegeneration [58]. Therefore, the inhibition of apoptotic cell death may be partly due to the inhibition of TLR4 and p-JNK. The effects of long-term oral administration on synaptic fucntion and memory were further evaluated; as hypothesized, the expression levels of synaptic markers were significantly upregulated in the ALA-cotreated mice brains compared to the Aβ-injected group. Overall, the demonstrated neuroprotective effects of omega-3 fatty acids against Aβ-induced neurodegeneration and memory impairment were in accordance with previously conducted studies [59,60].

Collectively, ALA may counteract Aβ-induced neuroinflammation by regulating certain endogenous antioxidant mechanisms, as mentioned previously in a study of diabetic rats [61]. However, we did not evaluate oxidative-stress-related effects in the treated groups in this study. The antioxidant effects of ALA may be responsible for the inhibition of amyloid-beta aggregation and neuronal loss; as suggested previously, the inhibition of oxidative stress is responsible for reducing amyloid-beta deposition and neuroinflammation [17]. Drawing from our results, we suggest that ALA may have multitargeted effects against Aβ-induced neurodegeneration. The main limitations of this study included the lack of study on a more mechanistic level and that the effects of ALA in transgenic mice were not evaluated; these areas need further analyses. Moreover, the ratio of serum neutrophils to lymphocytes should be evaluated, as this is a predictable marker of inflammation [62], and the effects of ALA should also be investigated in further studies.

## 5. Conclusions

In conclusion, ALA was demonstrated to have strong anti-neuroinflammatory, anti-amyloidogenic, and anti-apoptotic effects, and it was also shown to improve neuronal survival and memory deficits in the Aβ-infused mouse model. Based on our studies and previous reports, ALA may protect mice brains against Aβ-induced glial-cell-mediated neuronal cell loss and memory dysfunction. Our results may be helpful for the advancement of new therapeutic approaches for the management of AD-like conditions.
